# Association between systemic immune-inflammation index and risk of lower extremity deep venous thrombosis in hospitalized patients: a 10-year retrospective analysis

**DOI:** 10.3389/fcvm.2023.1211294

**Published:** 2023-06-16

**Authors:** Xi Chen, Yili Ou, Zhicong Wang, Hailong Liu, Yuehong Liu, Mozhen Liu

**Affiliations:** ^1^ Department of Orthopedics, People’s Hospital of Deyang City, Deyang, China; ^2^ Department of Orthopedics, The First Affiliated Hospital of Dalian Medical University, Dalian, China

**Keywords:** systemic immune-inflammation index, lower extremity deep venous thrombosis, inflammation, risk factor, biomarker

## Abstract

**Background:**

The systemic immune-inflammation index (SII), as a novel inflammatory biomarker, has recently attracted attention in cardiovascular disease research. However, the relationship between SII and risk of lower extremity deep venous thrombosis (LEDVT) remains unclear to date. Thus, this study aimed to explore the association in a large sample over a 10-year period (2012–2022).

**Methods:**

All hospitalized patients undergoing lower extremity compression ultrasonography (CUS) examination were consecutively screened by searching our hospital information system database. The receiver operating characteristic (ROC) curve analysis was used to identify the optimal cut-off value for high and low SII group. Multivariate logistic regression analyses were performed to investigate the relationship between SII and LEDVT risk. Propensity score matching (PSM), subgroup and sensitivity analyses were also conducted. Moreover, restricted cubic spline (RCS) regression and two-piecewise linear regression models were used to assess the dose-response relationship between natural log transformed SII [ln(SII)] and risk of LEDVT.

**Results:**

A total of 16,725 consecutive hospitalized patients were included, and 1,962 LEDVT events occurred. After adjusting for confounding factors, patients in the high SII group (≥ 574.2 × 10^9^/L) showed a 1.740-fold risk of LEDVT (95% *CI*: 1.546–1.959, *P* < 0.001), and elevated ln(SII) was associated with a 36.1% increased risk of LEDVT (95% *CI*: 1.278–1.449, *P* < 0.001). PSM, subgroup and sensitivity analyses confirmed the robustness of the association. A non-linear relationship was observed (*P*_non−linear _< 0.001), with a threshold value of 5.6 × 10^9^/L for all LEDVT events. Above the threshold, each unit increase in ln(SII) had a 1.369-fold higher risk of LEDVT (95% *CI*: 1.271–1.475, *P* < 0.001). The association also existed in both distal and proximal LEDVT.

**Conclusion:**

Elevated SII is significantly associated with an increased risk of LEDVT in hospitalized patients. Additionally, the association is non-linear and exhibit a threshold effect.

## 1. Introduction

Lower extremity deep venous thrombosis (LEDVT), which refers to thrombus formation in the deep venous system of the legs, is a global medical problem that affects approximately 1 per 1,000 individuals annually (1). As is well known, LEDVT is a major cause of post-thrombotic syndrome (PTS), fatal pulmonary embolism (PE) and even sudden death in hospitalized patients (2, 3). Until now, early diagnosis and timely treatment remain an effective strategy to reduce the risk of these complications (4, 5). However, the accurate diagnosis of LEDVT is challenging due to its unspecific symptoms and signs, potentially leading to misdiagnosis and incorrect treatment decisions (5). On the other hand, thrombotic events cannot be completely prevented even with effective antithrombotic drugs, implying that other underlying mechanisms may contribute to the therapeutic gap (6).

Recently, immune inflammatory system has been found to play a key role in DVT initiation and resolution (7–9). The systemic immune-inflammation index (SII), as an integrated indicator of local immune response and systemic inflammation, was significantly associated with cerebral venous thrombosis (10), portal venous thrombosis (11), PE (12), and other cardiovascular diseases (e.g., ischemic stroke, myocardial infarction, peripheral arterial disease) (13). Although SII was also considered to be a promising potential marker for DVT (14), existing evidences on the association between SII and LEDVT risk were particularly limited and conflicting (15–18). In these studies, most of the study populations were orthopedic patients [e.g., total knee arthroplasty (TKA), hip fracture, tibial plateau fracture], thus leaving a relative lack of evidence for generalization to other hospitalized patients (15, 16, 18). Furthermore, these studies were limited by their relatively small sample size, ranging from 273 to 1,179 patients (15–18). More importantly, a recent meta-analysis has systematically assessed the quality of current studies, and the certainty of the evidence was rated as very low using the Grading of Recommendations Assessment, Development, and Evaluation (GRADE) system (13). As a result, it is difficult to reach a consensus on the relationship between SII and LEDVT risk, thereby limiting its use in clinical practice (13, 14).

In the present study, we aimed to explore the association between SII and risk of LEDVT in a large sample, using our hospital information system (HIS) database over a 10-year period. To our knowledge, no previous study has evaluated the dose-response relationship between SII and LEDVT risk, in other words, whether this association is threshold or linear remains unclear. Therefore, we further examined the potential non-linear relationship for the association.

## 2. Method

### 2.1. Patients

**Figure 1:**
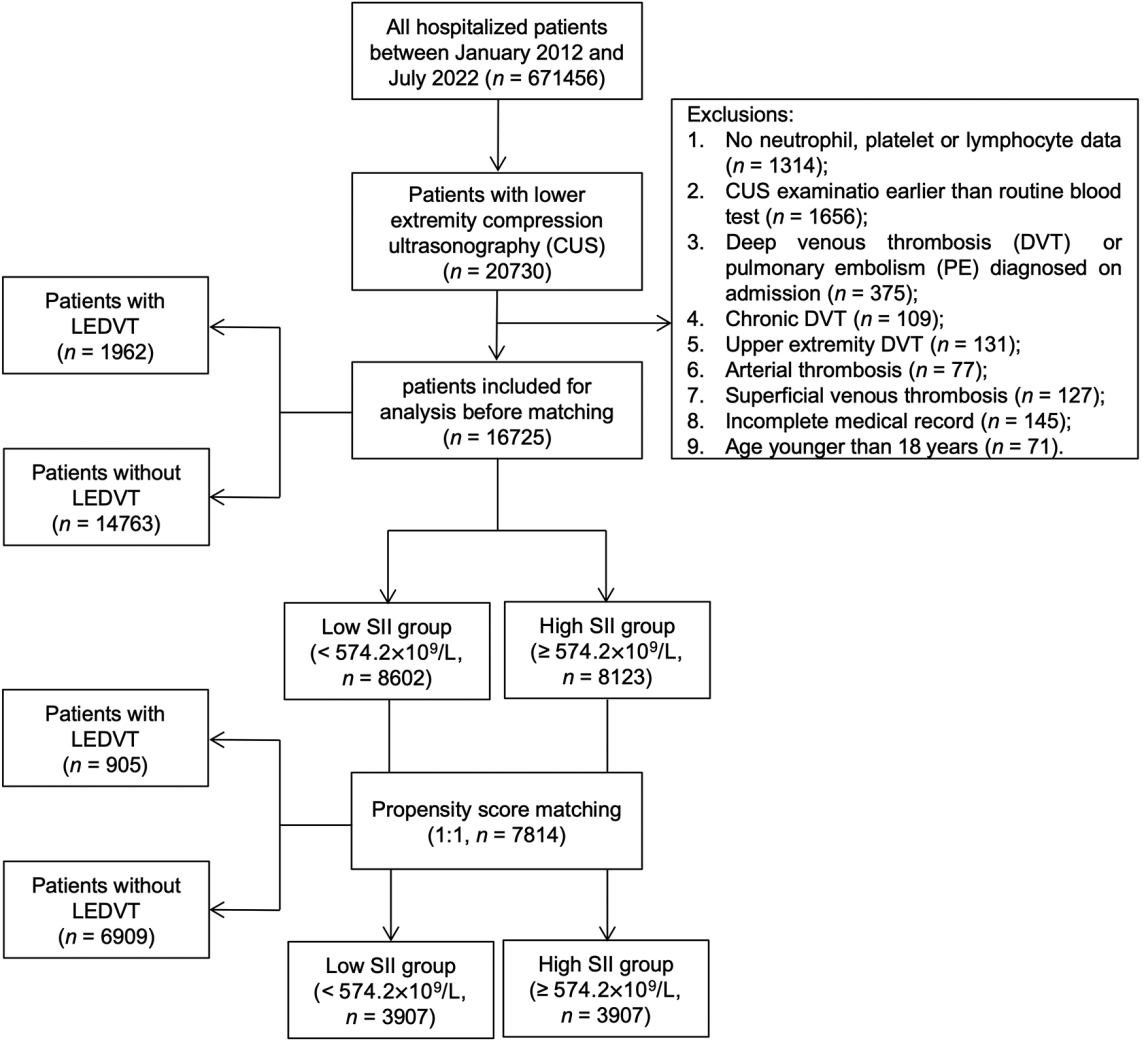
Flowchart of patient inclusion and exclusion. LEDVT, lower extremity deep venous thrombosis; SII, systemic immune-inflammation index.

This single-center, retrospective observational study was performed at a tertiary teaching hospital in China. The study protocol was reviewed and approved by the Institutional Ethics Committee of People's Hospital of Deyang City (No. 2021-04-019-K01), and the need for informed consent was waived owing to the retrospective nature of the study and anonymous data collection. In this study, all hospitalized patients between January 1, 2012 and July 31, 2022 were screened by searching the HIS database (*n* = 671,456), as described in our previous work (19). At the initial screening, 20,730 patients with lower extremity compression ultrasonography (CUS) were considered. After that, patients were excluded if they met any of the following criteria: (1) no neutrophil, platelet or lymphocyte data; (2) CUS examination earlier than routine blood test; (3) DVT or PE diagnosed on admission; (4) chronic DVT; (5) upper extremity DVT; (6) arterial thrombosis; (7) superficial venous thrombosis; (8) incomplete medical record; (9) age younger than 18 years. Finally, a total of 16,725 consecutive hospitalized patients were included in this study (Figure 1).

### 2.2. Data collection

The following patient characteristics were extracted from the electronic medical records: age, sex, body mass index (BMI), smoking and drinking history. According to the Working Group on Obesity in China, obesity was defined as BMI ≥ 28.0 kg/m^2^ (20). Smoking and drinking status were categorized into never, former or current. In accordance with a previous study (21), the International Classification of Diseases-10 (ICD-10) of discharge diagnoses were used to identify comorbidities: hypertension (codes: I10-I13, I15), diabetes mellitus (codes: E11-E14), chronic obstructive pulmonary disease (COPD, codes: J42-J44), atrial fibrillation (code: I48), heart failure (code: I50), stroke (codes: I60-I64), hepatic insufficiency (codes: K72.0-K72.1, K72.9), renal insufficiency (codes: N17-N19) and cancer (codes: C00-C97, D00-D09).

At our institute, blood samples were routinely drawn on the day of admission or the second day after admission. After blood drawing, routine blood tests were performed immediately using automatic hematology analyzers (Sysmex XN2000, Kobe, Japan). In this study, several hematological indicators closely related to DVT were collected, including white blood cell (WBC, reference range: 3.5–9.5 × 10^9^/L) (22), red blood cell (RBC, reference range: 3.8–5.1 × 10^9^/L) (23), and hemoglobin (reference range: 115.0–150.0 g/L) (24).

The SII was calculated as described in our previous study (25), using the following formula: SII = neutrophil count (reference range: 1.8–6.3 × 10^9^/L) × platelet count (reference range: 101.0–320.0 × 10^9^/L)/lymphocyte count (reference range: 1.1–3.2 × 10^9^/L). The unit of SII was expressed as 10^9^/L.

### 2.3. Study outcomes

As we described previously (26), lower extremity CUS was performed by an experienced radiologist, and the results were reviewed by another senior radiologist, using a color-coded ultrasound system with a 3–9 MHz linear-array transducer (iU 22, Philips Healthcare, Netherlands). The scanning was routinely performed at iliac vein, common femoral vein, superficial femoral vein, deep femoral vein, popliteal vein, anterior tibial vein, posterior tibial vein, fibular vein and calf muscle vein. DVT was diagnosed according to Robinov group's criteria (27). The primary outcome was the occurrence of all LEDVT events during the hospitalization, which was double confirmed by ultrasound reports and ICD-10 diagnoses of LEDVT (codes: I80.1, I80.2, I80.3, I80.8, I80.9, I82.8 and I82.9). Based on the thrombus location recorded in the ultrasound reports, proximal LEDVT was defined as thrombus occurring in the popliteal vein and/or above, whereas below the popliteal vein as distal LEDVT. Patients with both proximal and distal thrombus were regarded as proximal LEDVT (28). The secondary outcomes were the occurrence of distal and proximal LEDVT.

### 2.4. Statistical analyses

Prior to analysis, missing values were detected in all variables, with 30.45% missing in obesity (*n *= 5,093), 4.25% missing in smoking status (*n *= 710), and 25.72% missing in drinking status (*n *= 2,629). Given the large number of missing values, missing indicator method was used to handle these missing data, and coded as a separate category (unknown) (29).

Normality of continuous variables (SII, age, WBC, RBC, hemoglobin) were first checked by the Shapiro-Wilk test, showing that none of these variables conformed to the normal distribution. Continuous variables were described as median [interquartile range (IQR)], whereas the other categorical variables were reported as numbers (percentages). The receiver operating characteristic (ROC) curve analysis was used to determine the optimal cut-off value for high and low SII group. Differences between the two groups were compared using Wilcoxon rank-sum test or Pearson's chi-square test. Furthermore, SII data was natural log transformed [ln(SII)], and the differences between patients with and without LEDVT were visualized by box and whisker plots (Tukey method).

**Figure 2:**
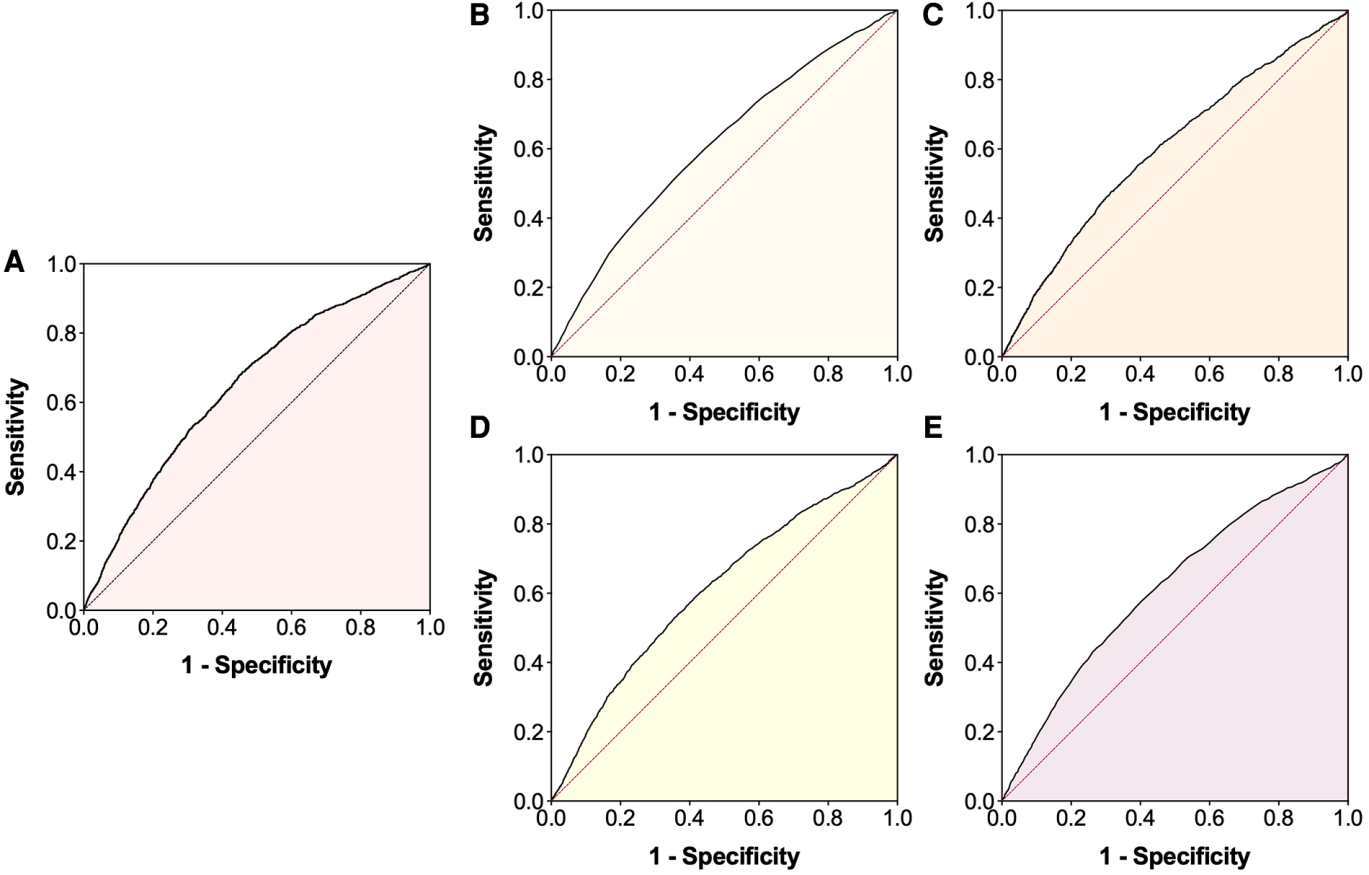
Receiver operating characteristic (ROC) curves for the occurrence of lower extremity deep venous thrombosis. (**A**) Systemic immune-inflammation index; (**B**) Age; (**C**) White blood cell; (**D**) Red blood cell; (**E**) Hemoglobin.

Before multivariate analyses, the linearity assumption for continuous variables was checked by the Box-Tidwell test (30), it was found to be violated for age, WBC, RBC and hemoglobin. For this reason, these continuous variables were converted to categorical variables based on the cut-off values by ROC curves (Supplementary Table S1, Figures 2B–E). Multicollinearity was assessed by the variance inflation factor (VIF), with a VIF ≥5 indicating multicollinearity (31). No significant multicollinearity was found between variables (Supplementary Table S2). Subsequently, multivariate logistic regression analyses were performed to evaluate the association between SII and LEDVT risk in different models. Model 1: SII data was entered as a categorical variable (high vs. low SII). Model 2: SII data was entered as a continuous variable [per ln(SII) increase]. Confounders variables that influences both the independent variable (Table 1) and dependent variable (Supplementary Table S3) were included into the multivariate logistic analyses, including age, sex, obesity, diabetes mellitus, COPD, atrial fibrillation, heart failure, stroke, hepatic insufficiency, renal insufficiency, WBC, RBC, and hemoglobin. Adjusted odds ratios (*OR*) with 95% confidence intervals (*CI*) were calculated.

**Table 1:** Baseline characteristics before and after propensity score matching.

Variables	All patients (*n* = 16,725)	Before matching (*n *= 16,725)				After matching (*n *= 7,814)			
		Low SII group (*n *= 8,602)	High SII group (*n *= 8,123)	SMD	*P value*	Low SII group (*n *= 3,907)	High SII group (*n *= 3,907)	SMD	*P value*
Age, year (median [IQR])	66.0 (55.0, 75.0)	65.0 (55.0, 73.0)	68.0 (56.0, 77.0)	0.118	<0.001	67.0 (57.0, 75.0)	67.0 (56.0, 76.0)	0.036	0.563
Men, *n* (%)	8,754 (52.3)	4,432 (51.5)	4,322 (53.2)	0.037	0.029	2,105 (53.9)	2,037 (52.1)	0.039	0.123
Obesity, *n* (%)	1,186 (7.1)	649 (7.5)	537 (6.6)	0.245	<0.001	280 (7.2)	279 (7.1)	0.033	0.341
Current smoking, *n* (%)	3,763 (22.5)	1,932 (22.5)	1,831 (22.5)	0.006	0.923	921 (23.6)	884 (22.6)	0.018	0.124
Current drinking, *n* (%)	4,597 (27.5)	2,398 (27.9)	2,199 (27.1)	0.012	0.473	1,105 (28.3)	1,074 (27.5)	0.005	0.681
**Comorbidities, *n* (%)**									
Hypertension	6,416 (38.4)	3,149 (36.6)	3,267 (40.2)	0.084	<0.001	1,671 (42.8)	1,603 (41.0)	0.039	0.119
Diabetes mellitus	6,510 (38.9)	3,777 (43.9)	2,733 (33.6)	0.239	<0.001	1,490 (38.1)	1,511 (38.7)	0.013	0.625
COPD	2,143 (12.8)	857 (10.0)	1,286 (15.8)	0.293	<0.001	535 (13.7)	518 (13.3)	0.021	0.573
Atrial fibrillation	806 (4.8)	336 (3.9)	470 (5.8)	0.228	<0.001	208 (5.3)	189 (4.8)	0.056	0.328
Heart failure	417 (2.5)	178 (2.1)	239 (2.9)	0.199	<0.001	115 (2.9)	97 (2.5)	0.097	0.21
Stroke	2,381 (14.2)	1,042 (12.1)	1,339 (16.5)	0.198	<0.001	656 (16.8)	638 (16.3)	0.018	0.584
Hepatic insufficiency	592 (3.5)	87 (6.86)	383 (3.75)	0.114	<0.001	127 (3.3)	137 (3.5)	0.043	0.531
Renal insufficiency	1,186 (7.1)	425 (4.9)	761 (9.4)	0.379	<0.001	307 (7.9)	299 (7.7)	0.016	0.735
Cancer	2,217 (13.3)	1,175 (13.7)	1,042 (12.8)	0.04	0.113	578 (14.8)	561 (14.4)	0.019	0.586
**Laboratory data, median (IQR)**									
WBC, 10^9^/L	6.61 (5.20, 8.72)	5.56 (4.58, 6.73)	8.35 (6.55, 10.87)	1.013	<0.001	6.58 (5.51, 7.73)	6.63 (5.54, 7.84)	0.003	0.153
RBC, 10^9^/L	4.20 (3.71, 4.63)	4.29 (3.86, 4.69)	4.08 (3.55, 4.56)	0.254	<0.001	4.23 (3.74, 4.65)	4.19 (3.69, 4.63)	0.014	0.359
Hemoglobin, g/L	127.0 (111.0, 140.0)	130.0 (117.0, 142.0)	122.0 (105.0, 136.0)	0.367	<0.001	126.0 (111.0, 139.0)	126.0 (110.0, 139.0)	0.022	0.614

SII, systemic immune-inflammation index; SMD, standardized mean difference; IQR, interquartile range; COPD, chronic obstructive pulmonary disease; WBC, white blood cell; RBC, red blood cell.

To further minimize the effect of potential confounders, a 1:1 propensity score matching (PSM) was performed between patients with high and low SII. Propensity scores for each patient were calculated using a multivariate logistic regression with the following variables: age, sex, obesity, smoking, drinking, hypertension, diabetes mellitus, COPD, atrial fibrillation, heart failure, stroke, hepatic insufficiency, renal insufficiency, cancer, WBC, RBC and hemoglobin. The greedy, nearest-neighbor method without replacement was used for matching, with a caliper width of 0.20 standard deviation (SD) of the logit of the propensity score (32). Standardized mean difference (SMD) was used to evaluate the balance of baseline characteristics before and after matching, with a SMD < 0.1 indicating adequate balance (33). As mentioned above, multivariate logistic regression analyses were also conducted in these matched samples.

Moreover, we examined the association in subgroups stratified by age, sex, obesity, smoking, drinking, diabetes mellitus, COPD, atrial fibrillation, heart failure, stroke, hepatic insufficiency, renal insufficiency, cancer, WBC, RBC and hemoglobin, because these factors were found to be associated with LEDVT (Supplementary Table S3). Considering the sample size of each subgroup, subgroup analyses were limited to the primary outcome only before matching, and adjusted for confounders variables except for the one defining the subgroup. To determine the effect of missing data, sensitivity analyses based on complete cases were also performed.

Finally, we applied restricted cubic spline (RCS) regressions with 5 knots to explore the dose-response relationship between ln(SII) and risk of LEDVT. Nonlinearity was tested with a likelihood ratio test comparing the spline model to a linear model. If the non-linear association was observed, a two-piecewise linear regression model was used to estimate the threshold effect of ln(SII) on LEDVT risk.

All reported *P* values are two-sided, and *P* < 0.05 was considered statistically significant. All analyses were conducted using JMP Pro software (version 16.0.0; SAS Institute Inc., Cary, NC, USA) and GraphPad Prism (version 9.1.1; GraphPad Software, San Diego, California, USA).

## 3. Results

### 3.1. Baseline characteristics

The baseline characteristics are presented in Table 1. Overall, the median age was 66.0 years, 52.3% were men, 7.1% were obesity, 22.5% were current smoking and 27.5% were current drinking. The most common comorbidities were diabetes mellitus (38.9%) and hypertension (38.4%). The median SII and ln(SII) values were 555.0 (318.6, 1,098.4) × 10^9^/L and 6.3 (5.8, 7.0) × 10^9^/L, respectively. Using the ROC curve analysis (Figure 2A), the optimal cut-off value was 574.2 × 10^9^/L for SII [sensitivity: 68.8%, specificity: 54.1%, area under curve (AUC): 0.647, 95% *CI*: 0.634–0.660]. The patients were then divided into low SII group (<574.2 × 10^9^/L, *n* = 8,602, 51.4%) and high SII group (≥574.2 × 10^9^/L, *n* = 8,123, 48.6%). Significant differences in most variables were observed between the two groups, except for smoking, drinking and cancer (Table 1).

### 3.2. LEDVT events

**Figure 3:**
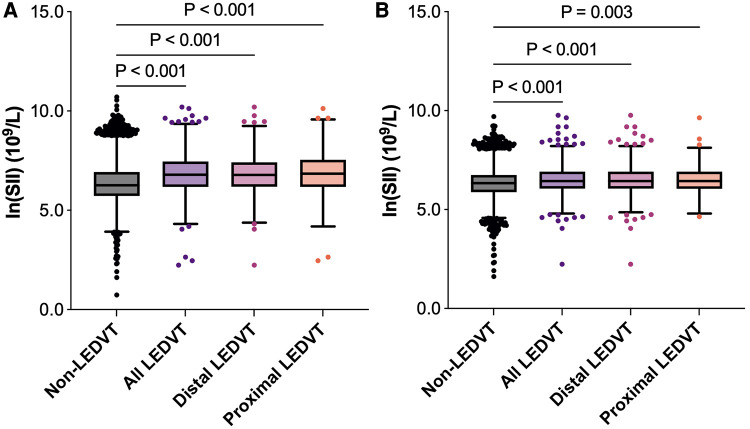
Box and whisker plots of ln(SII) in different groups. The box depicts the interquartile range (IQR), the horizontal line inside the box is the median, the whiskers extend to data points up to 1.5 × IQR, and dots are data points outside the whisker range. (**A**) Before matching. (**B**) After matching. SII, systemic immune-inflammation index; LEDVT, lower extremity deep venous thrombosis.

Of these patients, 1,962 (11.7%) were diagnosed with LEDVT and confirmed by CUS examinations. According to the thrombus location, 1,440 (73.4%) were distal LEDVT and 522 (26.6%) were proximal LEDVT. As shown in Figure 3A, patients with LEDVT had significant higher ln(SII) levels than those without (6.8 [6.2, 7.5] vs. 6.3 [5.7, 6.9] × 10^9^/L, *P *< 0.001). This trend was also seen in distal and proximal LEDVT (*P *< 0.001).

### 3.3. Association between SII and LEDVT before matching

**Figure 4:**
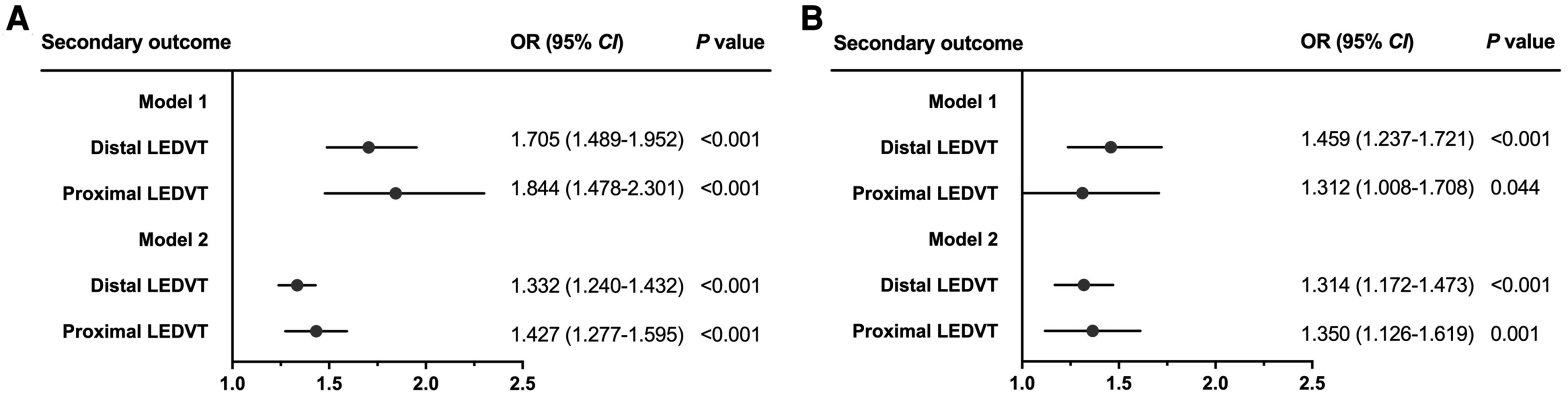
Forest plots for the association between SII and secondary outcome before matching (**A**) and after matching (**B**). Dots and horizontal lines represent adjusted odds ratio (OR) and 95% confidence interval (*CI*), respectively. Model 1: adjust for SII (categorical variable, ≥574.2 vs. <574.2 × 10^9^/L) and confounders variables, including age, sex, obesity, diabetes mellitus, chronic obstructive pulmonary disease, atrial fibrillation, heart failure, stroke, hepatic insufficiency, renal insufficiency, white blood cell, red blood cell, and hemoglobin. Model 2: adjust for SII [continuous variable, per ln (SII) increase] and confounders variables mentioned above. LEDVT, lower extremity deep venous thrombosis.

After adjusting for confounding factors (Table 2, Model 1), patients in the high SII group showed a 1.740-fold risk of LEDVT (95% *CI*: 1.546–1.959, *P* < 0.001). When SII was entered into the multivariate analysis as a continuous variable (Model 2), elevated ln(SII) still remained a significant risk factor (OR = 1.361, 95% *CI*: 1.278–1.449, *P* < 0.001). Also, this positive relationship existed in both distal and proximal LEDVT (Figure 4A). At the same time, age, sex, obesity, diabetes mellitus, atrial fibrillation, heart failure, stroke, hepatic insufficiency, WBC, RBC and hemoglobin were independently associated with a higher risk of LEDVT (Table 2, all *P* < 0.05).

**Table 2:** Multivariate logistic regression analyses of factors associated with lower extremity deep venous thrombosis before matching (*n *= 16,725).

Variables	Model 1		Model 2	
	OR (95% *CI*)	*P* value	OR (95% *CI*)	*P* value
SII	1.740 (1.546–1.959)	<0.001	1.361 (1.278–1.449)	<0.001
Age	1.635 (1.473–1.815)	<0.001	1.627 (1.466–1.807)	<0.001
Sex	1.169 (1.056–1.294)	0.003	1.172 (1.059–1.297)	0.003
Obesity	1.592 (1.293–1.959)	<0.001	1.622 (1.318–1.997)	<0.001
Diabetes mellitus	2.406 (2.137–2.708)	<0.001	2.392 (2.124–2.692)	<0.001
COPD	1.163 (1.016–1.331)	0.028	1.132 (0.988–1.296)	0.074
Atrial fibrillation	1.363 (1.124–1.653)	0.002	1.371 (1.130–1.662)	0.001
Heart failure	1.379 (1.055–1.801)	0.019	1.382 (1.057–1.805)	0.018
Stroke	1.202 (1.051–1.375)	0.007	1.219 (1.066–1.394)	0.004
Hepatic insufficiency	1.621 (1.299–2.021)	<0.001	1.576 (1.262–1.967)	<0.001
Renal insufficiency	1.005 (0.840–1.201)	0.959	1.005 (0.840–1.202)	0.959
White blood cell	1.413 (1.265–1.579)	<0.001	1.314 (1.167–1.480)	<0.001
Red blood cell	1.392 (1.206–1.608)	<0.001	1.387 (1.200–1.602)	<0.001
Hemoglobin	1.327 (1.147–1.536)	<0.001	1.318 (1.138–1.526)	<0.001

OR, odds ratio; CI, confidence interval; SII, systemic immune-inflammation index; COPD, chronic obstructive pulmonary disease.

Model 1: adjust for SII (categorical variable, ≥574.2 vs. <574.2 × 10^9^/L) and confounders variables, including age, sex, obesity, diabetes mellitus, chronic obstructive pulmonary disease, atrial fibrillation, heart failure, stroke, hepatic insufficiency, renal insufficiency, white blood cell, red blood cell, and hemoglobin.

Model 2: adjust for SII [continuous variable, per ln(SII) increase] and confounders variables mentioned above.

### 3.4. Association between SII and LEDVT after matching

After PSM, 7,814 patients were identified (3,907 in each group), and the baseline characteristics were well balanced between the two groups, with SMDs less than 0.1 for all variables (Table 1, Supplementary Figure S1). Among these patients, 905 (11.6%) had LEDVT, including 670 (74.0%) with distal LEDVT and 235 (26.0%) with proximal LEDVT. Similarly to the results before matching, patients with LEDVT exhibited higher ln(SII) levels than those without, and this difference remained significant in patients with distal and proximal LEDVT (Figure 3B). The results of multivariate analyses indicated that both high SII group (OR = 1.422, 95% *CI*: 1.232–1.641, *P* < 0.001; Table 3, Model 1) and elevated ln(SII) (OR = 1.326, 95% *CI*: 1.200–1.465, *P* < 0.001; Table 3, Model 2) were significantly associated with an increased risk of LEDVT. Moreover, the forest plot showed that high SII was an independent risk factor for both distal and proximal LEDVT (Figure 4B, *P* < 0.05).

**Table 3:** Multivariate logistic regression analyses of factors associated with lower extremity deep venous thrombosis after matching (*n *= 7,814).

Variables	Model 1		Model 2	
	OR (95% *CI*)	*P* value	OR (95% *CI*)	*P* value
SII	1.422 (1.232–1.641)	<0.001	1.326 (1.200–1.465)	<0.001
Age	1.740 (1.495–2.025)	<0.001	1.729 (1.485–2.013)	<0.001
Sex	1.209 (1.042–1.402)	0.012	1.211 (1.045–1.405)	0.011
Obesity	1.491 (1.096–2.028)	0.011	1.496 (1.099–2.034)	0.010
Diabetes mellitus	2.318 (1.956–2.747)	<0.001	2.290 (1.932–2.714)	<0.001
COPD	1.105 (0.905–1.349)	0.328	1.073 (0.878–1.310)	0.492
Atrial fibrillation	1.296 (0.977–1.719)	0.072	1.302 (0.982–1.728)	0.067
Heart failure	1.785 (1.245–2.559)	0.002	1.799 (1.254–2.581)	0.001
Stroke	1.108 (0.915–1.343)	0.292	1.102 (0.910–1.335)	0.319
Hepatic insufficiency	1.879 (1.368–2.580)	<0.001	1.833 (1.333–2,521)	<0.001
Renal insufficiency	1.023 (0.791–1.324)	0.860	1.005 (0.777–1.301)	0.968
White blood cell	1.252 (1.074–1.459)	0.004	1.169 (1.001–1.366)	0.048
Red blood cell	1.435 (1.166–1.766)	0.001	1.429 (1.161–1.760)	0.001
Hemoglobin	1.341 (1.087–1.653)	0.006	1.316 (1.067–1.623)	0.010

OR, odds ratio; CI, confidence interval; SII, systemic immune-inflammation index; COPD, chronic obstructive pulmonary disease.

Model 1: adjust for SII (categorical variable, ≥574.2 vs. <574.2 × 10^9^/L) and confounders variables, including age, sex, obesity, diabetes mellitus, chronic obstructive pulmonary disease, atrial fibrillation, heart failure, stroke, hepatic insufficiency, renal insufficiency, white blood cell, red blood cell, and hemoglobin.

Model 2: adjust for SII [continuous variable, per ln(SII) increase] and confounders variables mentioned above.

### 3.5. Subgroup analyses and sensitivity analyses

**Figure 5:**
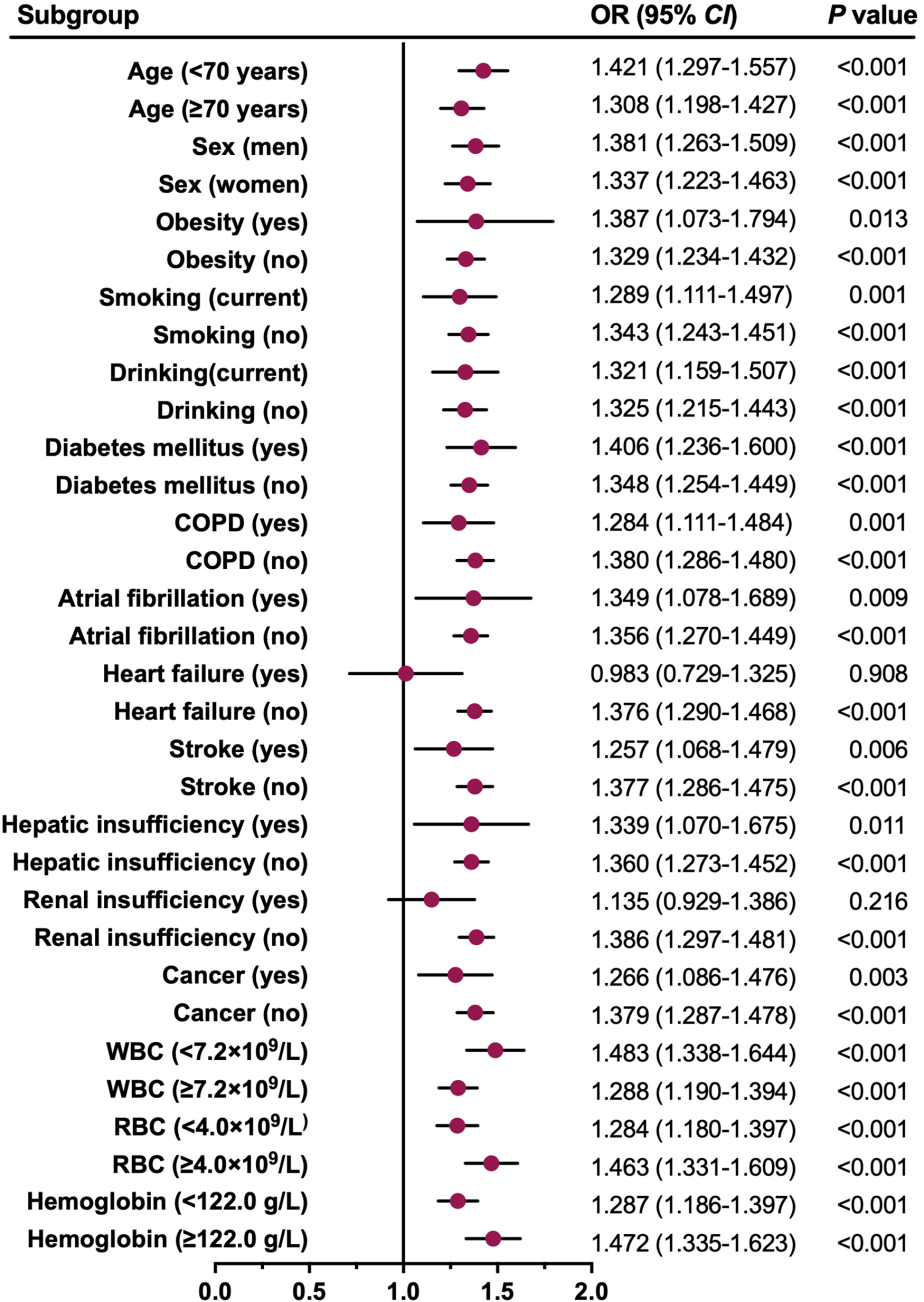
Forest plot for the association between ln(SII) and all LEDVT events in different subgroups. Dots and horizontal lines represent adjusted odds ratio (OR) and 95% confidence interval (*CI*), respectively. The associations (ORs) were adjusted for confounders variables except for the one defining the subgroup. COPD, chronic obstructive pulmonary disease; WBC, white blood cell; RBC, red blood cell.

To further verify the robustness of this association, a series of subgroup analyses were conducted, and the results are graphically presented using a forest plot (Figure 5). Consistent with the main findings, patients with elevated ln(SII) had significantly higher adjusted ORs for LEDVT in nearly all subgroups (OR range: 1.257–1.483, all *P* < 0.05), except for patients with heart failure (*P* = 0.908), and renal insufficiency (*P* = 0.216).

After excluding 7,040 patients with any missing data, a total of 9,685 patients (1,015 with distal LEDVT, 309 with proximal LEDVT) before matching, and 4,642 patients (475 with distal LEDVT, 138 with proximal LEDVT) after matching were available for further analyses. All sensitivity analyses yielded similar findings to the main analyses (Table 4).

**Table 4:** Sensitivity analyses of the association between ln(SII) and lower extremity deep venous thrombosis before and after matching.

Outcomes	Before matching (*n* = 9,685)		After matching (*n* = 4,642)	
	OR (95% *CI*)	*P* value	OR (95% *CI*)	*P* value
**Primary outcome**				
All LEDVT	1.315 (1.216–1.422)	<0.001	1.257 (1.110–1.423)	<0.001
**Secondary outcome**				
Distal LEDVT	1.289 (1.181–1.408)	<0.001	1.233 (1.073–1.417)	0.003
Proximal LEDVT	1.401 (1.208–1.624)	<0.001	1.344 (1.051–1.720)	0.019

OR, odds ratio; CI, confidence interval; SII, systemic immune-inflammation index; LEDVT, lower extremity deep venous thrombosis.

The associations (ORs) were adjusted for age, sex, obesity, diabetes mellitus, chronic obstructive pulmonary disease, atrial fibrillation, heart failure, stroke, hepatic insufficiency, renal insufficiency, white blood cell, red blood cell and hemoglobin.

### 3.6. Dose-response relationship between SII and LEDVT risk

**Figure 6:**
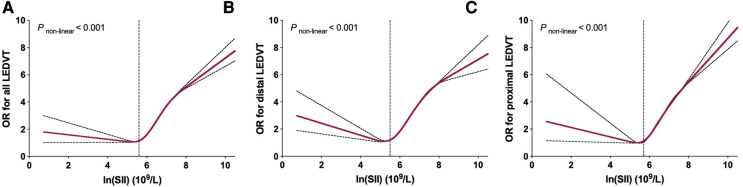
Restricted cubic spline plots between ln(SII) and multivariate adjusted odds ratio (OR) of LEDVT. (**A**) All LEDVT events. (**B**) Distal LEDVT. (**C**) Proximal LEDVT. The associations (ORs) were adjusted for ln(SII) and confounders variables, including age, sex, obesity, diabetes mellitus, chronic obstructive pulmonary disease, atrial fibrillation, heart failure, stroke, hepatic insufficiency, renal insufficiency, white blood cell, red blood cell, and hemoglobin.

As shown in Figure 6, significant non-linear relationships were observed between ln(SII) and risk of all, distal and proximal LEDVT events (*P* < 0.001). As ln(SII) increased, the risk of LEDVT first decreased, reached a minimum and then increased rapidly. Using two-piecewise linear regressions, the threshold values of ln(SII) were 5.6 × 10^9^/L for all LEDVT (SII = 270.4 × 10^9^/L), 5.5 × 10^9^/L for distal LEDVT (SII = 244.7 × 10^9^/L), and 5.7 × 10^9^/L for proximal LEDVT (SII = 298.9 × 10^9^/L). Below the threshold, increased ln(SII) was not associated with risk of LEDVT (Table 5, all *P* > 0.05). Nevertheless, above the threshold, each unit increase in ln(SII) had a 1.369-fold, 1.338-fold and 1.390-fold increased risk of all, distal and proximal LEDVT, respectively (*P* < 0.001).

**Table 5:** Threshold analyses for the association between ln (SII) and risk of LEDVT.

	All LEDVT		Distal LEDVT		Proximal LEDVT	
	OR (95% *CI*)	*P* value	OR (95% *CI*)	*P* value	OR (95% *CI*)	*P* value
Threshold value	5.6 × 10^9^/L		5.5 × 10^9^/L		5.7 × 10^9^/L	
<Threshold value	0.933 (0.661–1.317)	0.693	0.859 (0.523–1.411)	0.549	0.730 (0.449–1.187)	0.205
≥Threshold value	1.369 (1.271–1.475)	<0.001	1.338 (1.231–1.455)	<0.001	1.390 (1.220–1.584)	<0.001

SII, systemic immune-inflammation index; LEDVT, lower extremity deep venous thrombosis; OR, odds ratio; CI, confidence interval.

The associations (ORs) were adjusted for age, sex, obesity, diabetes mellitus, chronic obstructive pulmonary disease, atrial fibrillation, heart failure, stroke, hepatic insufficiency, renal insufficiency, white blood cell, red blood cell and hemoglobin.

## 4. Discussion

Thrombus formation is a complex process, which involves the interaction of a variety of blood cells, including neutrophils, platelets and lymphocytes (7, 8). Based on these blood cells, various immune-inflammation biomarkers were used as diagnostic and prognostic tools for DVT, such as neutrophil/lymphocyte ratio (NLR), and platelet/lymphocyte ratio (PLR) (14). The SII, which is a combination of neutrophils, platelets and lymphocytes, has emerged as a novel inflammation and immune marker. As expected, we found that increased SII (either as categorical or continuous variable) was significantly associated with a higher risk of LEDVT. This finding was consistent with studies from Melinte et al. (15), Zhang et al. (16), and Mureșan et al. (17), as described in Supplementary Table S4. However, Liu et al. (18) evaluated the relationship between SII and DVT risk in 1,179 patients with tibial plateau fracture, and SII was not independently associated with the occurrence of preoperative DVT, although univariate analysis reached statistical significance. The possible reason for this might be that multivariate analysis included the collinear variables (e.g., neutrophils, platelets, NLR, PLR), thereby affecting the result. To address this issue, we calculated the VIFs for each regression models, and VIFs between all variables were less than 2.3 (Supplementary Table S2), indicating that our results were not affected by the multicollinearity.

Distal and proximal LEDVT often differ substantially in terms of patient characteristics, risk factors and clinical management (34). For this reason, we further explored whether this relationship existed in both distal and proximal LEDVT. Fortunately, the results persisted even after full adjustment, further supporting the association between SII and LEDVT risk. Moreover, a series of PSM analyses, subgroup analyses and sensitivity analyses were performed to assess the robustness of this association. These analyses yielded similar findings, suggesting that our results were robust and reliable. As such, our study provided strong evidence for a positive link between systemic inflammation and thrombosis, implying that targeting this interface might be a promising strategy to prevent thrombosis and enhance the efficacy of anticoagulant therapy alone (6).

Furthermore, we observed a significant non-linear association between ln(SII) and LEDVT risk. As clearly shown in Figure 6, the risk of LEDVT first decreased and then increased rapidly with the rise of ln(SII). Although no previous study has investigated the dose-response relationship, similar non-linear associations were found between SII and risk of all-cause mortality (35, 36), arrhythmias (37), and rheumatoid arthritis (38). In these studies, the threshold values of ln(SII) were 5.97 × 10^9^/L (35), 6.57 × 10^9^/L (36), and 6.36 × 10^9^/L (38), respectively. Using two-piecewise linear regressions, the threshold values in this study were 5.6 × 10^9^/L for all LEDVT, 5.5 × 10^9^/L for distal LEDVT, and 5.7 × 10^9^/L for proximal LEDVT. Importantly, the risk of LEDVT increased rapidly after reaching the threshold value. For instance, each unit increase in ln(SII) above the threshold value was associated with a 1.369-fold increased risk of LEDVT. The SII is easily accessible, inexpensive, and feasible in routine clinical practice. Therefore, the threshold value of SII may be useful to identify hospitalized patients at high risk of LEDVT.

Notably, the diagnostic accuracy of SII for DVT was not high. In this study, the AUC was 0.647, with a sensitivity of 68.8% and specificity of 54.1%. Similarly, another two studies reported an AUC of 0.663 in elderly patients with hip fracture (16), and 0.605 in patients with tibial plateau fracture (18). In addition to its diagnostic role, a recent meta-analysis also showed that the pooled AUC, sensitivity and specificity were 0.63, 0.68 and 0.55, concluding that SII had a certain prognostic value for overall survival in patients with nasopharyngeal carcinoma (39). Due to its insufficient accuracy and complexity of thrombus formation, SII alone is not sufficient to diagnose LEDVT, and other clinical indicators need to be combined to improve the diagnostic ability. Future studies are thus required to explore whether the combination of SII with other biomarkers may provide additional diagnostic value in predicting DVT.

However, certain limitations should also be considered. First, this is an observational study and can only demonstrate association and not causation. At the same time, due to the retrospective nature of the study design, some important risk factors related to DVT could not be accessed, such as pregnancy status, hormone replacement therapy, prior history of venous thromboembolism (VTE), and antithrombotic therapy before admission. Additionally, antithrombotic drugs after LEDVT diagnosis were not obtained due to lack of detailed medication regimen in the database (19). Further prospective studies are still needed to confirm our findings. Second, the study population was restricted to hospitalized patients, and derived from a single academic medical center. This may limit generalization of these results. Third, the SII value is dynamic, and its dynamic status has been found to be associated with risk of cardiovascular diseases (40). In this study, we only used the SII data on admission, which may lead to underestimate this association (35). Fourth, all LEDVT events in this study were diagnosed by ultrasonography, which may lead to a lower accuracy than venography (26). Finally, although we carefully adjusted for potential confounders, biases resulting from unknown and unmeasured confounders may still exist (37).

In conclusion, elevated SII is significantly associated with an increased risk of LEDVT in hospitalized patients. Additionally, the association is non-linear and exhibit a threshold effect.

## Data Availability

The raw data supporting the conclusions of this article will be made available by the authors, without undue reservation.

## References

[1] Chopard R Albertsen IE Piazza G. Diagnosis and treatment of lower extremity venous thromboembolism: a review. JAMA. (2020) 324(17):1765–76. 10.1001/jama.2020.17272 33141212

[2] Cosmi B Stanek A Kozak M Wennberg PW Kolluri R Righini M The post-thrombotic syndrome-prevention and treatment: VAS-European independent foundation in angiology/vascular medicine position paper. Front Cardiovasc Med. (2022) 9:762443. 10.3389/fcvm.2022.762443 35282358 PMC8907532

[3] Dubois-Silva Á Barbagelata-López C Piñeiro-Parga P López-Jiménez L Riera-Mestre A Schellong S Deep vein thrombosis symptoms and 30-day mortality in acute pulmonary embolism. Eur J Intern Med. (2023) 108:43–51. 10.1016/j.ejim.2022.11.007 36400669

[4] Rognoni C Lugli M Maleti O Tarricone R. Clinical guidelines versus current clinical practice for the management of deep vein thrombosis. J Vasc Surg Venous Lymphat Disord. (2021) 9(5):1334–1344.e1. 10.1016/j.jvsv.2021.01.020 33744498

[5] Bhatt M Braun C Patel P Patel P Begum H Wiercioch W Mustafa: diagnosis of deep vein thrombosis of the lower extremity: a systematic review and meta-analysis of test accuracy. Blood Adv. (2020) 4(7):1250–64. 10.1182/bloodadvances.2019000960 32227213 PMC7160276

[6] Stark K Massberg S. Interplay between inflammation and thrombosis in cardiovascular pathology. Nat Rev Cardiol. (2021) 18(9):666–82. 10.1038/s41569-021-00552-1 33958774 PMC8100938

[7] Budnik I Brill A. Immune factors in deep vein thrombosis initiation. Trends Immunol. (2018) 39(8):610–23. 10.1016/j.it.2018.04.010 29776849 PMC6065414

[8] Mukhopadhyay S Johnson TA Duru N Buzza MS Pawar NR Sarkar R Fibrinolysis and inflammation in venous thrombus resolution. Front Immunol. (2019) 10:1348. 10.3389/fimmu.2019.01348 31258531 PMC6587539

[9] Nicklas JM Gordon AE Henke PK. Resolution of deep venous thrombosis: proposed immune paradigms. Int J Mol Sci. (2020) 21(6):2080. 10.3390/ijms21062080 32197363 PMC7139924

[10] Ding J Song B Xie X Li X Chen Z Wang Z Inflammation in cerebral venous thrombosis. Front Immunol. (2022) 13:833490. 10.3389/fimmu.2022.833490 35444662 PMC9013750

[11] Xing Y Tian Z Jiang Y Guan G Niu Q Sun X A practical nomogram based on systemic inflammatory markers for predicting portal vein thrombosis in patients with liver cirrhosis. Ann Med. (2022) 54(1):302–9. 10.1080/07853890.2022.2028893 35060835 PMC8786242

[12] Kantarcioglu B Darki A Siddiqui F Krupa E Vural M Kacmaz M Predictive role of blood cellular indices and their relationship with endogenous glycosaminoglycans as determinants of inflammatory biomarkers in pulmonary embolism. Clin Appl Thromb Hemost. (2022) 28:10760296221104801. 10.1177/10760296221104801 35733366 PMC9234831

[13] Ye Z Hu T Wang J Xiao R Liao X Liu M Systemic immune-inflammation index as a potential biomarker of cardiovascular diseases: a systematic review and meta-analysis. Front Cardiovasc Med. (2022) 9:933913. 10.3389/fcvm.2022.933913 36003917 PMC9393310

[14] Xue J Ma D Jiang J Liu Y. Diagnostic and prognostic value of immune/inflammation biomarkers for venous thromboembolism: is it reliable for clinical practice? J Inflamm Res. (2021) 14:5059–77. 10.2147/jir.S327014 34629886 PMC8494998

[15] Melinte RM Arbănași EM Blesneac A Zolog DN Kaller R Mureșan AV Inflammatory biomarkers as prognostic factors of acute deep vein thrombosis following the total knee arthroplasty. Medicina. (2022) 58(10):1502. 10.3390/medicina58101502 36295662 PMC9608310

[16] Zhang L He M Jia W Xie W Song Y Wang H Analysis of high-risk factors for preoperative DVT in elderly patients with simple hip fractures and construction of a nomogram prediction model. BMC Musculoskelet Disord. (2022) 23(1):441. 10.1186/s12891-022-05377-8 35546231 PMC9092837

[17] Mureșan AV Hălmaciu I Arbănași EM Kaller R Arbănași EM Budișcă OA Prognostic nutritional index, controlling nutritional Status (CONUT) score, and inflammatory biomarkers as predictors of deep vein thrombosis, acute pulmonary embolism, and mortality in COVID-19 patients. Diagnostics. (2022) 12(11):2757. 10.3390/diagnostics12112757 PMC9689150 36428817

[18] Liu D Zhu Y Chen W Li J Zhao K Zhang J Relationship between the inflammation/immune indexes and deep venous thrombosis (DVT) incidence rate following tibial plateau fractures. J Orthop Surg Res. (2020) 15(1):241. 10.1186/s13018-020-01765-9 32616051 PMC7331237

[19] Jianjun Z Yan C Zhicong W Xi C Yuehong L Mozhen L. Anatomic distribution of lower extremity deep venous thrombosis is associated with an increased risk of pulmonary embolism: a 10-year retrospective analysis. Front Cardiovasc Med. (2023) 10:1154875. 10.3389/fcvm.2023.1154875 37034353 PMC10073460

[20] Zhou B. Predictive values of body mass index and waist circumference to risk factors of related diseases in Chinese adult population. Zhonghua Liu Xing Bing Xue Za Zhi. (2002) 23(1):5–10. 10.3760/j.issn:0254-6450.2002.01.003 12015100

[21] Glise Sandblad K Rosengren A Sörbo J Jern S Hansson PO. Pulmonary embolism and deep vein thrombosis-comorbidities and temporary provoking factors in a register-based study of 1.48 million people. Res Pract Thromb Haemost. (2022) 6(4):e12714. 10.1002/rth2.12714 35677029 PMC9166387

[22] Wang G Zhao W Zhao Z Wang D Wang D Bai R Leukocyte as an independent predictor of lower-extremity deep venous thrombosis in elderly patients with primary intracerebral hemorrhage. Front Neurol. (2022) 13:899849. 10.3389/fneur.2022.899849 35903126 PMC9314880

[23] Liu X Li T Xu H Wang C Ma X Huang H Hyperglycemia may increase deep vein thrombosis in trauma patients with lower limb fracture. Front Cardiovasc Med. (2022) 9:944506. 10.3389/fcvm.2022.944506 36158801 PMC9498976

[24] Xiong X Li T Yu S Cheng B. Association between red blood cell indices and preoperative deep vein thrombosis in patients undergoing total joint arthroplasty: a retrospective study. Clin Appl Thromb Hemost. (2022) 28:10760296221149029. 10.1177/10760296221149029 36572965 PMC9806375

[25] Wang Z Jiang W Chen X Yang L Wang H Liu Y. Systemic immune-inflammation index independently predicts poor survival of older adults with hip fracture: a prospective cohort study. BMC Geriatr. (2021) 21(1):155. 10.1186/s12877-021-02102-3 33663402 PMC7934427

[26] Wang Z Chen X Wu J Zhou Q Liu H Wu Y Low mean platelet volume is associated with deep vein thrombosis in older patients with hip fracture. Clin Appl Thromb Hemost. (2022) 28:10760296221078837. 10.1177/10760296221078837 35157546 PMC8848069

[27] Rabinov K Paulin S. Roentgen diagnosis of venous thrombosis in the leg. Arch Surg. (1972) 104(2):134–44. 10.1001/archsurg.1972.04180020014004 5008903

[28] Ma J Du P Qin J Zhou Y Liang N Hu J Incidence and risk factors predicting deep venous thrombosis of lower extremity following spinal fractures. Sci Rep. (2021) 11(1):2441. 10.1038/s41598-021-82147-x 33510388 PMC7843965

[29] Choi J Dekkers OM le Cessie S. A comparison of different methods to handle missing data in the context of propensity score analysis. Eur J Epidemiol. (2019) 34(1):23–36. 10.1007/s10654-018-0447-z 30341708 PMC6325992

[30] Thompson WK Xie M White HR. Transformations of covariates for longitudinal data. Biostatistics. (2003) 4(3):353–64. 10.1093/biostatistics/4.3.353 12925503

[31] Kim JH. Multicollinearity and misleading statistical results. Korean J Anesthesiol. (2019) 72(6):558–69. 10.4097/kja.19087 31304696 PMC6900425

[32] Wang Y Cai H Li C Jiang Z Wang L Song J Optimal caliper width for propensity score matching of three treatment groups: a monte carlo study. PLoS One. (2013) 8(12):e81045. 10.1371/journal.pone.0081045 24349029 PMC3859481

[33] Zhang Z Kim HJ Lonjon G Zhu Y. Balance diagnostics after propensity score matching. Ann Transl Med. (2019) 7(1):16. 10.21037/atm.2018.12.10 30788363 PMC6351359

[34] Schellong S Ageno W Casella IB Chee KH Schulman S Singer DE Profile of patients with isolated distal deep vein thrombosis versus proximal deep vein thrombosis or pulmonary embolism: RE-COVERY DVT/PE study. Semin Thromb Hemost. (2022) 48(4):446–58. 10.1055/s-0041-1729169 33971682 PMC9246482

[35] Cao Y Li P Zhang Y Qiu M Li J Ma S Association of systemic immune inflammatory index with all-cause and cause-specific mortality in hypertensive individuals: results from NHANES. Front Immunol. (2023) 14:1087345. 10.3389/fimmu.2023.1087345 36817427 PMC9932782

[36] He L Xie X Xue J Xie H Zhang Y. Association of the systemic immune-inflammation index with all-cause mortality in patients with arteriosclerotic cardiovascular disease. Front Cardiovasc Med. (2022) 9:952953. 10.3389/fcvm.2022.952953 36172591 PMC9510918

[37] Yang X Zhao S Wang S Cao X Xu Y Yan M Systemic inflammation indicators and risk of incident arrhythmias in 478,524 individuals: evidence from the UK biobank cohort. BMC Med. (2023) 21(1):76. 10.1186/s12916-023-02770-5 36855116 PMC9976398

[38] Liu B Wang J Li YY Li KP Zhang Q. The association between systemic immune-inflammation index and rheumatoid arthritis: evidence from NHANES 1999–2018. Arthritis Res Ther. (2023) 25(1):34. 10.1186/s13075-023-03018-6 36871051 PMC9985219

[39] Wang L Qin X Zhang Y Xue S Song X. The prognostic predictive value of systemic immune index and systemic inflammatory response index in nasopharyngeal carcinoma: a systematic review and meta-analysis. Front Oncol. (2023) 13:1006233. 10.3389/fonc.2023.1006233 36816962 PMC9936064

[40] Li J He D Yu J Chen S Wu Q Cheng Z Dynamic status of SII and SIRI alters the risk of cardiovascular diseases: evidence from kailuan cohort study. J Inflamm Res. (2022) 15:5945–57. 10.2147/jir.S378309 36274831 PMC9584782

