# Supplementary material for: Association between systemic immune-inflammation index and risk of lower extremity deep venous thrombosis in hospitalized patients: a 10-year retrospective analysis
Source: Front Cardiovasc Med. 2023 Jun 16;10:1211294. doi: 10.3389/fcvm.2023.1211294 (PMC10313113; doi:10.3389/fcvm.2023.1211294)
Supplement: Supplementary file 1 [file Table1.docx]

**Supplemental Table 1** Receiver operating characteristic (ROC) analysis for the occurrence of lower extremity deep venous thrombosis.

| **Variables** | **Cut-off value** | **Sensitivity (%)** | **Specificity (%)** | **AUC**  **(95% *CI*)** | **Low grade, *n* (%)** | **High grade, (%)** |
| --- | --- | --- | --- | --- | --- | --- |
| Age | 70.0 | 53.8 | 62.1 | 0.609 (0.595-0.622) | 10073 (60.2) | 6652 (39.8) |
| WBC | 7.8 | 47.6 | 68.4 | 0.600 (0.586-0.613) | 9831 (58.8) | 6894 (41.2) |
| RBC | 4.0 | 54.1 | 63.2 | 0.610 (0.597-0.624) | 6398 (38.3) | 10327(61.7) |
| Hemoglobin | 122.0 | 57.7 | 59.7 | 0.615 (0.602-0.628) | 6780 (40.5) | 9945 (59.5) |

AUC, area under curve; CI, confidence interval; WBC, white blood cell; RBC, red blood cell.
