# Supplementary material for: Association between systemic immune-inflammation index and risk of lower extremity deep venous thrombosis in hospitalized patients: a 10-year retrospective analysis
Source: Front Cardiovasc Med. 2023 Jun 16;10:1211294. doi: 10.3389/fcvm.2023.1211294 (PMC10313113; doi:10.3389/fcvm.2023.1211294)
Supplement: Supplementary file 2 [file Table2.docx]

**Supplemental Table 2 Multicollinearity analysis with variance inflation factors for multivariate logistic analysis.**

| **Variables** | **Before matching** | |  | **After matching** | |
| --- | --- | --- | --- | --- | --- |
|  | **Model 1** | **Model 2** |  | **Model 1** | **Model 2** |
| SII | 1.394 | 1.544 |  | 1.001 | 1.028 |
| Age | 1.134 | 1.135 |  | 1.123 | 1.123 |
| Sex | 1.069 | 1.069 |  | 1.074 | 1.074 |
| Obesity | 2.206 | 2.207 |  | 2.139 | 2.139 |
| Diabetes mellitus | 1.082 | 1.084 |  | 1.081 | 1.081 |
| COPD | 1.089 | 1.092 |  | 1.084 | 1.086 |
| Atrial fibrillation | 1.092 | 1.092 |  | 1.081 | 1.082 |
| Heart failure | 1.079 | 1.079 |  | 1.079 | 1.079 |
| Stroke | 1.062 | 1.061 |  | 1.052 | 1.052 |
| Hepatic insufficiency | 1.018 | 1.018 |  | 1.017 | 1.017 |
| Renal insufficiency | 1.095 | 1.096 |  | 1.089 | 1.090 |
| White blood cell | 1.321 | 1.448 |  | 1.059 | 1.083 |
| Red blood cell | 2.195 | 2.194 |  | 2.121 | 2.121 |
| Hemoglobin | 2.254 | 2.254 |  | 2.154 | 2.157 |

SII, systemic immune-inflammation index; COPD, chronic obstructive pulmonary disease.

Model 1: adjust for SII (categorical variable, ≥ 574.2 *vs.* < 574.2 × 10^9^/L) and confounders variables, including age, sex, obesity, diabetes mellitus, chronic obstructive pulmonary disease, atrial fibrillation, heart failure, stroke, hepatic insufficiency, renal insufficiency, white blood cell, red blood cell, and hemoglobin.

Model 2: adjust for SII (continuous variable, per ln[SII] increase) and confounders variables mentioned above.
