# Supplementary material for: Association between systemic immune-inflammation index and risk of lower extremity deep venous thrombosis in hospitalized patients: a 10-year retrospective analysis
Source: Front Cardiovasc Med. 2023 Jun 16;10:1211294. doi: 10.3389/fcvm.2023.1211294 (PMC10313113; doi:10.3389/fcvm.2023.1211294)
Supplement: Supplementary file 3 [file Table3.docx]

**Supplemental Table 3 Univariate logistic regression analyses of factors associated with lower extremity deep venous thrombosis before matching.**

| **Variables** | **OR (95% *CI*)** | ***P* value** |
| --- | --- | --- |
| Age | 1.905 (1.733-2.094) | <0.001 |
| Sex | 1.214 (1.105-1.334) | <0.001 |
| Obesity | 1.789 (1.465-2.185) | <0.001 |
| Current smoking | 1.418 (1.251-1.606) | <0.001 |
| Current drinking | 1.336 (1.189-1.501) | <0.001 |
| Hypertension | 1.024 (0.929-1.128) | 0.633 |
| Diabetes mellitus | 2.621 (2.341-2.935) | <0.001 |
| COPD | 1.636 (1.444-1.853) | <0.001 |
| Atrial fibrillation | 2.176 (1.827-2.592) | <0.001 |
| Heart failure | 1.908 (1.493-2.438) | <0.001 |
| Stroke | 1.372 (1.212-1.554) | <0.001 |
| Hepatic insufficiency | 1.844 (1.494-2.274) | <0.001 |
| Renal insufficiency | 1.411 (1.196-1.665) | <0.001 |
| Cancer | 1.356 (1.193-1.542) | <0.001 |
| White blood cell | 1.911 (1.738-2.102) | <0.001 |
| Red blood cell | 2.013 (1.831-2.213) | <0.001 |
| Hemoglobin | 2.012 (1.830-2.213) | <0.001 |

OR, odds ratio; CI, confidence interval; COPD, chronic obstructive pulmonary disease.
