# Supplementary material for: Association between systemic immune-inflammation index and risk of lower extremity deep venous thrombosis in hospitalized patients: a 10-year retrospective analysis
Source: Front Cardiovasc Med. 2023 Jun 16;10:1211294. doi: 10.3389/fcvm.2023.1211294 (PMC10313113; doi:10.3389/fcvm.2023.1211294)
Supplement: Supplementary file 4 [file Table4.docx]

**Supplemental Table 4** Characteristics of studies investigating the association between systemic immune-inflammation index and deep venous thrombosis.

| **Study** | **Country** | **Study design** | **Sample Size** | **Patients** | **SII cut-off (**× 10^9^/L**)** | **Sensitivity (%)** | **Specificity (%)** | **AUC** | **Multivariate analysis outcome** |
| --- | --- | --- | --- | --- | --- | --- | --- | --- | --- |
| Melinte et al, 2022 [15] | Romania | Retrospective cohort case-control study | 273 | Patients undergoing total knee arthroplasty (TKA) | 1133.8 | 82.1 | 80.4 | 0.870 | Higher SII was an independent predictor of DVT (OR = 18.87, *P* < 0.001). |
| Zhang et al, 2022 [16] | China | Retrospective case-control study | 274 | Elderly patients with hip fracture | 1225.8 | 43.3 | 86.0 | 0.663 | Higher SII was an independent risk factor for preoperative DVT (OR = 1.002, *P* = 0.042). |
| Mureșan et al, 2022 [17] | Romania | Retrospective cohort study | 889 | COVID-19 patients | 1890.5 | 79.1 | 71.2 | 0.805 | Higher SII at admission strongly predict DVT risk (OR = 9.33, *P* < 0.001). |
| Liu et al, 2020 [18] | China | Retrospective case-control study | 1179 | Patients with tibial plateau fracture | 1066 | ND | ND | 0.605 | SII was not independently associated with preoperative DVT (OR: ND). |
| Our study, 2023 | China | Retrospective cohort study | 16725 | All hospitalized patients | 574.2 | 68.8 | 54.1 | 0.647 | Above the threshold value, elevated ln(SII) was significantly associated with an increased risk of LEDVT (OR = 1.369, *P* < 0.001). |

SII, systemic immune-inflammation index; AUC, area under curve; DVT, deep venous thrombosis; ND, no data; LEDVT, lower extremity deep venous thrombosis.
