# Supplementary figures and images for: Association between systemic immune-inflammation index and risk of lower extremity deep venous thrombosis in hospitalized patients: a 10-year retrospective analysis
Source: Front Cardiovasc Med. 2023 Jun 16;10:1211294. doi: 10.3389/fcvm.2023.1211294 (PMC10313113; doi:10.3389/fcvm.2023.1211294)

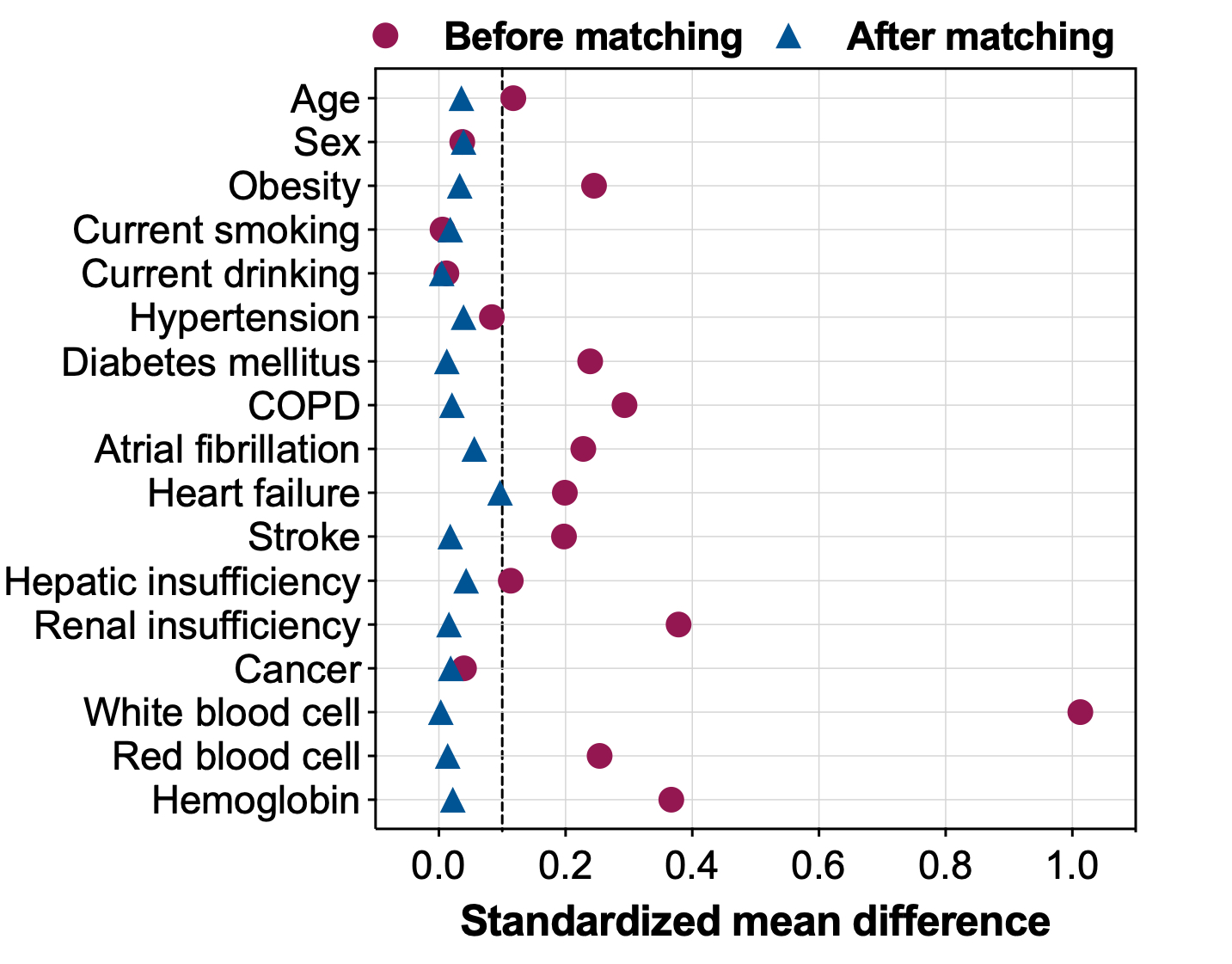

Supplement: Supplementary file 5 [file Image1.jpeg]
